# Advancing personalised oncologic care for older adults with head and neck cancer through structured international collaboration

**DOI:** 10.3389/fonc.2026.1858873

**Published:** 2026-07-27

**Authors:** Colm Mac Eochagain, Petr Szturz, Colm Mac Eochagain, Petr Szturz, Pierluigi Bonomo, Zsuzsanna Iyizoba, Paul Nankivell, Alexander Rühle, Yuki Saito, Kathrin Scheckenbach, Purnima Sundaresan, Ana Varges Gomes, Sue Yom, Paolo Bossi, Hisham Mehanna, Jan B. Vermorken

**Affiliations:** 1 Department of Medical Oncology, Trinity St James’ Cancer Institute, Dublin, Ireland; 2 Department of Oncology, University of Lausanne (UNIL) and Lausanne University Hospital (CHUV), Lausanne, Switzerland

**Keywords:** squamous cell carcinoma of head and neck, geriatric assessment, geriatric oncology, frailty, chemoradiotherapy, precision medicine, quality of life

## Introduction

Head and neck squamous cell carcinoma (HNSCC) encompasses malignancies arising from the mucosal surfaces of the oral cavity, oropharynx, hypopharynx, and larynx. Collectively, HNSCC is the eighth most common cancer worldwide, with GLOBOCAN 2022 estimating approximately 770,000 new cases and 384,000 global annual deaths (1). Incidence is projected to rise substantially over the coming decades, driven both by population aging and increasing rates of human papillomavirus (HPV)-associated oropharyngeal cancer in many high-income countries (2).

In Western populations, the majority of HNSCC diagnoses occur in patients aged 65 years or older, and this proportion is expected to grow, in both relative and absolute terms, as life expectancy increases and the population ages (3). Although older adults already comprise a majority of patients diagnosed with HNSCC, this patient population remains substantially under-represented and under-reported in the clinical trials that define standards of care (4–6).

From a biological standpoint, the efficacy of standard anticancer treatments in HNSCC, including surgery, radiotherapy, and systemic therapy, is generally preserved in selected fit older adults. However, treatment is more frequently complicated by reduced physiological reserve, comorbidity, nutritional compromise, impaired functional and cognitive status, and polypharmacy, all of which increase the risk of treatment-related toxicity and competing mortality (7). Patients with HNSCC carry a greater comorbidity burden at diagnosis than those with most other solid tumours, second only to lung cancer in a large prospective cross-cancer comparison (8), in part reflecting the strong association with tobacco and alcohol use (9) among those with HPV-negative disease. Treatment of HNSCC also places substantial demands on basic physical functions including swallowing, speech, and airway patency, and successful treatment, whether curative or palliative, relies heavily on coordinated nutritional, rehabilitative, and psychosocial support. Taken together, these factors make older adults with HNSCC a particularly important population in which to advance personalised oncogeriatric care.

The treatment landscape for HNSCC has evolved considerably in recent years. Immune checkpoint inhibitors are now established in recurrent and/or metastatic disease, with pembrolizumab (alone, or combined with platinum-based chemotherapy) as a first-line standard (10). More recently, the KEYNOTE-689 trial of perioperative pembrolizumab demonstrated significantly improved event-free survival in patients with resectable stage III-IVA HNSCC and stage III HPV-positive oropharyngeal carcinoma (11). Similarly, the NIVOPOSTOP trial showed improved disease-free survival when nivolumab was added to adjuvant cisplatin-based chemoradiotherapy and continued as maintenance for up to 6 months in resected high-risk HNSCC (12). However, the applicability of these findings to older adults, particularly frail or comorbid older adults, remains uncertain. Trial populations in these studies were predominantly younger and fitter, with good performance status (ECOG 0-1) and limited comorbidity; NIVOPOSTOP excluded patients aged 75 and above. As with a large majority of recent registration studies in oncology, these trials did not conduct geriatric assessments or apply geriatric-specific quality-of-life instruments, limiting our understanding of how these data apply to this population (5) (**Table 1**). In this Perspective, we outline the principal unmet needs in the management of older adults with HNSCC and propose a structured, internationally coordinated research agenda to address them.

**Table 1:** Age-related characteristics of selected landmark trials in mucosal HNSCC.

Trial (Year)	Setting	Median age, years (range)	% aged ≥65	Upper age limit	Performance status eligibility	Age-subgroup primary-endpoint efficacy reported (HR)
EXTREME NCT00122460 (2008) (44)	1st Line R/M	CT+Cetux:56 (NR); CT alone: 57 (NR)	17.4	None	KPS ≥70	OS <65yr: 0·74 (0·59–0·94) ≥65yr: 1·07 (0·65–1·77)
CHECKMATE-141 NCT02105636 (2016) (45)	1st+ Line R/M	Nivo: 59 (29–83); Control: 61 (28–78)	31.3	None	ECOG-PS 0-1	OS <65yr: 0·64 (0·45–0·89) ≥65yr <75 yr: 0·93 (0·56–1·54) ≥75yr: NR
KEYNOTE-040 NCT02252042 (2019) (46)	1st+ Line R/M	Pembro: 60 (19–85); SOC 60 (34-78)	32.9	None	ECOG-PS 0-1	OS <65yr: 0·94 (0·73–1·20); ≥65yr <75yr: 0·57 (0·37–0·87); ≥75yr: 1·13 (0·42–3·02)
KEYNOTE-048 NCT02358031 (2019) (10)	1st Line R/M	Total: 61 (20–94)	36.0	None	ECOG-PS 0-1	OS Pembro: <65yr: 0·82 (0·66–1·02) ≥65yr: 0·79 (0·59–1·05) Pembro+CT: <65yr: 0·83 (0·67–1·04) ≥65yr: 0·52 (0·38–0·71)
TPExtreme NCT02268695 (2021) (47)	1st Line R/M	TPEx: 60 (NR) EXTREME: 60 (NR)	NR	70	ECOG-PS 0-1	OS <60yr: 0·81 (0·62–1·07) ≥60yr: 0·97 (0·74–1·26)
KEYNOTE-689 NCT03765918 (2025) (11)	Perioperative LA-HNSCC	Pembro: 60.0 (29–82); Control: 61.0 (22–87)	33.3	None	ECOG-PS 0-1	EFS <65yr: 0·68 (0·51–0·90) ≥65yr: 0·83 (0·57–1·22)
NIVOPOSTOP NCT03576417 (2026) (12)	Adjuvant high-risk resected LA-HNSCC	Nivo+Cis+RT: 59 (18–74) Cis+RT: 59 (22–74);	22.7	74	ECOG-PS 0-1	DFS <65yr: 0·71 (0·54–0·95) ≥65yr: 0·95 (0·56–1·59)

Cetux, Cetuximab; Cis, Cisplatin; CT, Chemotherapy; DFS, Disease-Free Survival; ECOG, Eastern Cooperative Oncology Group Performance Status; EFS, Event-Free Survival; HR, Hazard Ratio; KPS, Karnofsky Performance Status; LA-HNSCC, Locally Advanced Head and Neck Squamous Cell Carcinoma; NCT, National Clinical Trial (Registration Number); NR, Not reported; Nivo, Nivolumab; OS, Overall Survival; Pembro, Pembrolizumab; R/M, Recurrent/Metastatic; RT, Radiotherapy; SOC, Standard of Care; Yr, Years.

## Unmet needs in the management of older adults with HNSCC

Many areas of uncertainty remain in the management of older adults with HNSCC. There is currently no broadly accepted consensus on how to define an “older” patient with HNSCC. Although 65 years has been considered a conventional threshold adopted by major international societies, the pivotal Meta-Analysis of Chemotherapy in Head and Neck cancer (MACH-NC) (13) used a cutoff of 70 years, and subgroup reporting across other studies is variable. The higher threshold may better reflect the changing demographic structure of Western populations, with increasing life expectancy and overall quality of life. However, the use of a fixed chronological cutoff fails to capture the substantial geographic and socioeconomic variability in the correlation between chronological and biological age, as well as health-adjusted life expectancy (14).

Although geriatric screening tools, including Geriatric-8 (G8), appear to have strong prognostic value and have been specifically validated in head and neck cancer populations, where they predict treatment complications and survival (15, 16), routine implementation of geriatric screening or comprehensive geriatric assessment in head and neck oncology practice appears to be limited, often because of time constraints, complexity, and limited availability of oncogeriatricians or other qualified personnel (17), and the extent to which geriatric assessment findings influence treatment decisions in this setting remains uncertain (18–22).

Beyond patient selection, the adaptation of treatment in frail or older patients (across all modalities: surgery, radiotherapy, systemic therapy) remains poorly standardised. For instance, the addition of chemotherapy to radiotherapy in older patients with locally advanced disease remains contentious. The MACH-NC demonstrated no clear survival benefit from concurrent chemotherapy beyond 70 years, but the authors noted that the proportion of competing mortality increased progressively with advancing age, potentially reflecting comorbidities or chemotherapy-related toxicity rather than a lack of efficacy (13). Moreover, numerous retrospective analyses suggest that carefully selected patients with good performance status may still benefit from this approach in terms of antitumour activity (23–25). Thus, a plausible conclusion is that older patients, albeit at higher risk of complications, may still derive a measurable efficacy benefit from the combined nonsurgical approach. In clinical practice, approaches vary, although compared with the situation two or more decades ago, physicians appear increasingly aware that strict chronological age cutoffs are not optimal, particularly in fit individuals, and that functional assessment and comorbidity profiling offer a more comprehensive and appropriate framework for treatment decision-making (17, 26, 27). This applies both to the three-weekly cisplatin schedule and to alternative schedules, including the use of alternative radiosensitisers (carboplatin, cetuximab, docetaxel, combination of low-dose carboplatin and paclitaxel) or treatment schedules (i.e. the use of weekly cisplatin), all of which are commonly used but none of which has been prospectively validated in a dedicated elderly HNSCC population (28–30). In the locally advanced setting, treatment guidelines and trial eligibility criteria are often framed around the concept of cisplatin ineligibility, a category that captures many older patients, but which is defined by organ dysfunction and comorbidity rather than geriatric frailty (28). Conflating the two may group patients with potentially different risk profiles to the same treatment strategy. Prospective trials with frailty-based eligibility criteria are needed to address this.

In the recurrent and metastatic setting, the ELAN programme demonstrated that geriatric assessment can meaningfully stratify treatment benefit, using a tool specific to HNSCC to classify patients aged 70 and older as fit or unfit and allocating them to distinct trials accordingly (31, 32). ELAN-FIT, a single-arm, phase II study, met its co-primary endpoints, with an objective response rate of 40% and a morbidity rate of 31% in fit older patients. The phase III randomised ELAN-UNFIT study found no benefit of cetuximab over methotrexate in frail patients (median overall survival 4.6 months in both arms). The applicability of the ELAN programme to current practice is nonetheless limited, since both trials were designed before checkpoint inhibitors entered first-line treatment, and their comparators no longer reflect the standard of care for combined positive score (CPS) positive disease. In FRAIL-IMMUNE (33) (GORTEC 2018-03, single-arm phase II), first-line durvalumab with weekly carboplatin/paclitaxel showed encouraging activity and tolerability; grade 3 or higher adverse events were frequent but predominantly haematological, and treatment-related deaths were uncommon. Among the subgroup of patients with an ECOG performance status of 2, the objective response rate was 60.5%, and toxicity led to permanent discontinuation of chemotherapy in 22.5% and of durvalumab in 7.5%. Beyond these trials, specific prospective data to guide systemic treatment decisions in older or frail patients with recurrent or metastatic HNSCC remain scarce.

From a surgical perspective, thresholds for major radical resections and microvascular free flap reconstruction in older patients differ between institutions, with limited prospective data to guide decisions. While free flap success rates appear comparable across age groups, frailty, rather than chronological age, is a predictor of postoperative complications, prolonged length of stay, and delayed adjuvant treatment delivery (34–36).

Similarly, hypofractionated radiotherapy regimens and elective nodal volume reduction are increasingly discussed as strategies to reduce treatment burden in older patients. Early prospective data are emerging, but a wide variety of schedules are in use and specific criteria for recommending these approaches have not yet been formalised (37–39). Approaches to supportive care, including nutritional intervention, prehabilitation, speech and swallowing therapy, and toxicity management, also vary between institutions, and the extent to which these services are available to and tailored for older patients is also poorly characterised.

Finally, traditional oncological endpoints may not fully capture what matters to older patients. Quality of life, functional preservation, treatment completion, maintenance of independence and an approach based on patients preferences may be more relevant to many patients than traditional time-to-event outcomes, including overall survival, yet these endpoints are not routinely collected or reported in existing studies (40–42). The joint ESMO/SIOG (European Society for Medical Oncology/International Society of Geriatric Oncology) Cancer in the Elderly Working Group has recently emphasised the need for trials to incorporate patient-centred endpoints, including composite measures of overall treatment utility and time to functional decline, alongside traditional survival outcomes (43).

## Establishing a framework for international collaborative research

Addressing these uncertainties in the management of this large patient population requires a collaborative, international approach. The management of older adults with HNSCC varies not only between individual clinicians but also across institutions, disciplines, and healthcare systems. A systematic mapping of current practice is a necessary first step both for understanding the scale and nature of variation, and for informing the design of future studies. Without detailed understanding of how older patients are currently assessed and treated across different settings, designing trials with appropriate objectives, interventions, endpoints, and eligibility criteria is challenging. Equally, future trials will only succeed if they are acceptable both to the clinicians who recruit to them and to the patients who enrol in them.

It is equally important to distinguish those questions that require prospective trial-level evidence from those that can be addressed through retrospective analyses or structured expert consensus, and making this distinction explicit is itself a valuable output of an international collaborative process, and one that can help direct limited research resources toward the areas of greatest need.

Some questions involve uncertainty about the risk-benefit of an intervention in older patients, for instance, the approach to systemic therapy for unfit patients with recurrent and/or metastatic disease in the immunotherapy era, the role of hypofractionated radiotherapy regimens and elective nodal volume reduction, and whether geriatric assessment-directed care improves outcomes compared to standard practice in this disease. Evaluations of quality of life may benefit from greater specificity for this population. These questions, among others, likely require prospective collaborative trials. Recently, the ESMO/SIOG working group has highlighted the value of pragmatic and adaptive trial designs, geriatric assessment-stratified approaches such as those used in the ELAN programme, and simplified geriatric data collection tools such as the G-CODE and American Society of Clinical Oncology (ASCO) Practical Geriatric Assessment, as strategies to make such trials feasible in this population (43).

Other questions relate to interventions that already exist but whose application in older patients is unstandardised, including the selection of patients for perioperative immunotherapy, criteria for cisplatin ineligibility in the absence of major organ dysfunction, thresholds for radical surgery and free flap reconstruction, and the choice and timing of geriatric screening. These issues may be better addressed through structured expert consensus.

## A structured research agenda for older adults

The Head and Neck Cancer International Group (HNCIG) is a collaborative network comprising over 20 national and regional cooperative groups from Europe, North America, Asia-Pacific, and South America, with a record of facilitating large-scale international studies, real-world data initiatives, and consensus projects. Its Older Adults Workstream was recently established to address the challenges of managing older patients with HNSCC, and has developed the Evaluating Practice in Oncogeriatric Care for Head and Neck cancer (EPOCH-HN) initiative. Head and neck cancer, as a disease that disproportionately affects older adults and whose management spans multiple disciplines and treatment modalities, is well suited as a model for advancing personalised oncologic care in this population through such international collaboration.

Addressing the uncertainties outlined above requires a multilayered programme of work, incorporating practice characterisation, evidence synthesis, consensus development, retrospective studies, and ultimately prospective trials, with each component building on the next. Through EPOCH-HN, we propose a structured research agenda comprising: narrative and systematic evidence reviews to frame key clinical questions and identify research priorities; an international practice pattern survey to quantify variation across disciplines and regions and to understand how clinicians address evidence gaps in day-to-day practice; and a Delphi-based consensus statement to provide practical guidance where trial-level evidence is lacking. Findings from earlier phases will directly inform the scope of later ones, ensuring that the evidence synthesis and consensus process address the questions that matter most in current clinical practice. The principal domains of unmet need and the corresponding contributions of the EPOCH-HN initiative are summarised in **Table 2**.

**Table 2:** Key domains of unmet need in the management of older adults with HNSCC and corresponding EPOCH-HN contributions.

	Unmet need	Evidence gap	EPOCH-HN contribution
1	No accepted definition of the “older” HNSCC patient	Thresholds vary (65 vs 70 years); fixed chronological cutoffs miss biological-age and geographic variability	Evidence review; Delphi consensus
2	Inconsistent use of geriatric screening and assessment in practice	Validated tools underused; uncertain influence on treatment decisions	Practice pattern survey; Delphi consensus
3	Poorly standardised treatment adaptation in older or frail patients across surgery, radiotherapy, and systemic therapy	Widely used regimens, schedules, and surgical thresholds never prospectively validated in a dedicated older population; frailty, not age, predicts complications	Evidence review; practice pattern survey; Delphi consensus; informing future retrospective analyses, future trials
4	“Cisplatin ineligibility” conflated with geriatric frailty	Eligibility framed by organ dysfunction and comorbidity rather than frailty	Practice pattern survey; consensus on selection criteria
5	Systemic therapy in recurrent/metastatic disease in the immunotherapy era	ELAN predates immunotherapy; FRAIL-IMMUNE limited to single-arm phase 2; other prospective data scarce	Evidence review; consensus; informing future prospective trial design
6	Patient-centred and functional endpoints under-collected	Quality of life, functional independence, and treatment completion rarely collected or reported	Trial-design standards advocacy; informing future trial design

In addition, the EPOCH-HN initiative enables a seamless transition into clinical research by supporting and informing future retrospective studies evaluating widely used but insufficiently studied treatment approaches, as well as prospective trials focusing on novel agents, quality of life, and geriatric interventions, incorporating the pragmatic, geriatric assessment-stratified designs advocated by the ESMO/SIOG working group.

## Conclusion

Older adults with HNSCC represent a large, growing, and underserved population. Older adults bear a disproportionate share of disease burden, yet the evidence guiding their treatment is largely extrapolated from trials enrolling younger and fitter patients. Clinical practice varies widely, geriatric assessment is inconsistently applied, and there is no international consensus on optimal management. As the population continues to age and as new treatments including perioperative immunotherapy and novel systemic agents introduce additional questions about patient selection, dosing, and treatment sequencing in older adults, the disparity between clinical need and available evidence will only grow.

A coordinated international effort is required to define standards of care, prioritise research questions, and to develop clinical practice recommendations that reflect the complexity of managing this patient population. The structured, multilayered approach described here, encompassing narrative review, evidence synthesis, practice pattern survey, consensus development, retrospective analysis, and, ultimately, prospective trials, provides a practical roadmap.

Critically, this roadmap must incorporate patient-centred endpoints and pragmatic trial designs, ensuring that future research captures not only antitumour activity and survival but also functional independence, quality of life, patients’ preferences and treatment tolerability, which are often more meaningful endpoints for older patients. The EPOCH-HN initiative will deliver three linked outputs in 2027/28: a synthesis of the existing evidence to define research priorities, an international practice pattern survey to characterise current variation in the care of older adults with HNSCC, and a Delphi-based consensus statement to provide practical guidance where trial-level evidence is lacking. Together, these will establish the evidence base and clinical priorities needed to direct future retrospective and prospective research toward the questions of greatest need.

## Group members of EPOCH-HN Working Group

Colm Mac Eochagain, Petr Szturz, Pierluigi Bonomo, Zsuzsanna Iyizoba, Paul Nankivell, Alexander Rühle, Yuki Saito, Kathrin Scheckenbach, Purnima Sundaresan, Ana Varges Gomes, Sue Yom, Paolo Bossi, Hisham Mehanna, and Jan B. Vermorken.
